# Predictors of lower extremity amputation in patients with diabetic foot ulcer: findings from MEDFUN, a multi-center observational study

**DOI:** 10.1186/s13047-019-0345-y

**Published:** 2019-06-14

**Authors:** Ejiofor Ugwu, Olufunmilayo Adeleye, Ibrahim Gezawa, Innocent Okpe, Marcelina Enamino, Ignatius Ezeani

**Affiliations:** 1 Department of Medicine, Division of Endocrinology, Diabetes and Metabolism, Enugu State University of Science and Technology, Enugu, Nigeria; 2 Department of Medicine, Division of Endocrinology, Diabetes and Metabolism, Lagos State University, Lagos, Nigeria; 3 Department of Medicine, Division of endocrinology, Diabetes and Metabolism, Bayero University, Kano, Nigeria; 4 Department of Medicine, Division of Endocrinology, Diabetes and Metabolism, Ahmadu Bello University, Zaria, Nigeria; 5 Department of Medicine, Division of Endocrinology, Diabetes and Metabolism, Federal Medical Center, Keffi, Nigeria; 6 Department of Medicine, Division of Endocrinology, Diabetes and Metabolism, Federal Medical Center, Umuahia, Nigeria

**Keywords:** Diabetes, Foot ulcer, Risk factors, Amputation, Predictors, MEDFUN, Nigeria, Africa

## Abstract

**Background:**

Lower extremity amputation (LEA) is a potential sequelae of diabetic foot ulceration (DFU) and is associated with huge morbidly and mortality. Low and middle income countries are currently at the greatest risk of diabetes-related complications and deaths. We sought to identify demographic, clinical and laboratory variables that significantly predict LEA in patients hospitalized for DFU.

**Methods:**

The Multi-center Evaluation of Diabetic Foot Ulcer in Nigeria (MEDFUN) was an observational study conducted between March 2016 and April 2017 in six tertiary healthcare institutions. We prospectively followed 336 diabetic patients hospitalized for DFU and managed by a multidisciplinary team until discharge or death. Demographic and diabetes-related information and ulcer characteristics were documented. Patients were evaluated for neuropathy, peripheral arterial disease (PAD) and medical co-morbidities while relevant laboratory and imaging tests were performed. The study end-points were ulcer healing, LEA, duration of hospitalization and mortality. Here we present data on amputation.

**Results:**

One hundred and nineteen subjects (35.4%) underwent LEA during the follow-up period. Univariate predictors of LEA were ulcer duration more than 1 month prior to hospitalization (*P* <  0.001), PAD (*P* <  0.001), Wagner grade ≥ 4 (*P* <  0.001), wound infection (P 0.041), Proteinuria (P 0.021), leucocytosis (P 0.001) and osteomyelitis (*P* <  0.001). On multivariate regression, only three variables emerged as significant independent predictors of LEA and these include: ulcer duration more than 1 month (O.R. 10.3, 95% C.I. 4.055–26.132), PAD (O.R. 2.8, 95% C.I. 1.520–5.110) and presence of osteomyelitis (O.R. 5.6, 95% C.I. 2.930–10.776). Age, gender, diabetes type and duration, neuropathy, glycemic control and anemia did not predict LEA in the studied population.

**Conclusion:**

We identified duration of ulcer greater than 1 month, PAD, Wagner grade 4 or higher, proteinuria, leucocytosis, wound infection and osteomyelitis as the significant predictors of LEA in patients hospitalized for DFU. Prompt attention to these risk factors may reduce amputation rate among these patients.

## Background

As a result of the relentless increase in the prevalence of diabetes mellitus (DM) in Africa, a corresponding rise in diabetes complications is expected. The International Diabetes Federation and World Health Organization jointly warn that these complications, if unchecked would threaten the viability of many African nations [1]. With diabetes prevalence of 5.7%, Nigeria is currently home to about 5 million adults living with diabetes. This appears to be a tip of the iceberg as it is estimated that about two-thirds of diabetes cases in Nigeria are yet undiagnosed [2]. Consequent upon this high prevalence of chronic undetected hyperglycemia, many individuals with diabetes present with already established chronic complications at the time of diagnosis [3].

One potentially preventable complication of diabetes that is associated with high morbidity and mortality is diabetic foot ulcer (DFU). It is estimated that a person with diabetes has up to 25% chance of developing DFU in his/her lifetime [4]. The burden of DFU is high both in Africa generally [5], and in Nigeria in particular [6, 7]. A recent update suggests that nearly 2 out of every 10 out-patients with diabetes in Nigeria have diabetic foot disease [7], and DFU accounts for nearly a third of diabetes-related hospital admissions [8]. Diabetic foot ulcer is associated with prolonged hospital stay, substantial economic burden and high mortality [9, 10]. Perhaps the most unpleasant potential consequence of DFU besides death is lower extremity amputation (LEA).

More than three-quarters of all LEAs performed in people with diabetes is secondary to DFU which is currently the leading cause of non-trauma related LEA globally [11]. The negative medical and psychosocial consequences of LEA in people with DM are substantial. About 10% of those who suffer major LEAs die intra-admission [12]. And post LEA survivors have a significantly reduced quality of life and higher risk of depression which may be related to impaired psychosocial functioning [13]. The long term prognosis after DFU-related LEA is also reportedly abysmal, with 3-year mortality after diabetic foot amputation ranging from 35 to 50% [14]. In fact, long term prognosis after major LEAs in people with diabetes has been shown to be comparable to breast and prostate malignancies in females and males respectively [15].

Diabetes-related LEA rates have significantly declined in many Western countries [16, 17]. This is however not the case in many parts of Africa where DFU-related LEA rates are still very high [5]. Less than half a decade ago, amputation rate as high as 52% was reported among patients hospitalized for DFU in one tertiary healthcare center in Nigeria [6]. Efforts to prevent this unpleasant scenario therefore deserve utmost attention, and this could be partly accomplished by risk factor identification. Regrettably, not much has been done in this direction in Nigeria. This research represents an effort to fill this very important gap.

## Methods

The Multi-center Evaluation of Diabetic Foot Ulcer in Nigeria (MEDFUN) was a 1 year prospective observational study conducted in six tertiary healthcare institutions across Nigeria, from March 2016 to April 2017. The centers included Enugu State University Teaching Hospital, Lagos State University Teaching Hospital, Aminu Kano Teaching Hospital, Ahmadu Bello University Teaching Hospital Zaria, Federal Medical Center Keffi and Federal Medical Center Umuahia. The inclusion criterion was all consenting adults ≥18 years with type 1 or type 2 diabetes hospitalized for DFU over the study period. Approval of the study protocol was obtained from the local Research and Ethics committee of each of the hospitals while verbal informed consent was obtained from each patient prior to recruitment.

Details of the methodology of the MEDFUN study have been published [18]. Summarily, we obtained relevant socio-demographic and diabetes-related information including gender, age, occupation, cigarette smoking status, diabetes type and duration. Distinction between type 1 and type 2 DM was made clinically based on combined parameters of age and method of diabetes control. Subjects who had been exclusively controlled on insulin since the time of diagnosis were classified as type 1 diabetes (T1DM) while those who were being treated with oral anti-diabetic drugs (OAD) with or without insulin were adjudged to have type 2 diabetes mellitus (T2DM).

Participants’ knowledge of proper foot care practices was assessed. This entailed inquiries about bare-foot walking, daily foot inspection, proper foot wear, use of emollients to prevent dryness of the feet, proper pedicure practice and early treatment of foot problems. They were also asked whether they had received any foot care education prior to foot ulceration. History of development and progression of ulcer including mechanism of ulceration, site of ulcer, duration of ulcer and prior ulcer treatment methods were also assessed. The severity of ulcer was graded using the Wagner grading system. This widely used DFU grading system, although has some limitations including not taking into account, wound size and vascular integrity of the foot, has the advantages of simplicity and ease of bedside application compared to newer wound classification systems. It does not require sophisticated laboratory or imaging tests and is therefore especially suitable for developing countries like ours with constrained resources. Besides, the Wagner grading system has been shown to have strong correlations with DFU outcomes in several studies. Clinical wound infection was determined according to the International Working Group on Diabetic Foot guideline (4) by the presence of purulent exudates or any two or more of the following: periwound edema, periwound redness, local warmth, foul smell, pain or tenderness on palpation and fever. Commonly known risk factors for DFU were also evaluated, including history of previous DFU, barefoot walking, improper foot wear, visual impairment, foot deformity, peripheral neuropathy and peripheral artery disease (PAD). Peripheral neuropathy was diagnosed by loss of pressure perception to Semmes-Weinstein 10 g monofilament test or diminished vibration sense using the 128 Hz tunning fork. Peripheral artery disease was suspected in the presence of diminished or impalpable dorsalis pedis and/or posterior tibial artery pulsations on manual examination, and then confirmed by the presence of significant arterial narrowing (> 50%) on Doppler ultrasonography of the lower limbs.

Relevant laboratory and imaging studies were performed for each subject including urine protein, complete blood count, glycated hemoglobin (HbA1c), blood culture, ulcer specimen culture, lipid profile, plain radiograph of the foot and Doppler ultrasonography of both lower limbs. Co-morbid complications including hypertension, anemia, shock, hyperglycemic emergency, hypoglycemia, stroke, kidney disease and cardiac failure were explored and documented.

Every patient received appropriate multi-disciplinary care including bed rest, wound debridement, daily wound dressing, antibiotic therapy, skin grafting and limited amputation in addition to control of blood glucose and treatment of associated co-morbidities. Follow up was continued until the patient was discharged from the hospital or exited by death. Outcome variables of interest included ulcer healing, amputation, duration of hospitalization and mortality. We defined amputation above the mid-tarsal bone or involving the big toe as major amputation, otherwise it was considered as minor amputation. There are no generally accepted criteria for classifying amputation as major or minor. Our definition was therefore arbitrary and based on the potential physical limitations imposed by amputation of or above the big toe as the latter is responsible for 40% of weight bearing on the toes, and also the last part of the foot to push off the ground during walking. At the end of subject recruitment, records of medical admissions over the study period were retrospectively reviewed in all the centers to determine the total number of medical admissions and diabetes-related admissions. Descriptive data of the entire study population have been published [18]. We hereby present results of sub-analysis of data related to amputation.

### Statistical analysis

For the current sub-analysis, we performed unadjusted associations between demographic, clinical and laboratory variables and amputation (whether major or minor) using the Chi-Square statistics for categorical variables and t-test for continuous variables. To identify independent predictors of amputation, we first performed univariate logistic regressions for each variable with amputation as the dependent outcome, and calculated variable odds ratios (ORs) and 95% confidence intervals (CI). All the variables that emerged as significant predictors at this univariate level of analysis were then simultaneously entered into a multivariate regression model that was reduced using a backward selection method. Model reliability was determined by the Hosmer and Lemeshow test of goodness of fit. Analysis was done with the Statistical Package for Social Sciences (IBM version 23.0; SPSS Inc., Chicago, IL, USA). All tests were 2-tailed and *P* <  0.05 was considered significant.

## Results

One hundred and nineteen (35.4%) out of the 336 subjects that participated in this study underwent LEA and 75.6% of these were major amputations. Table 1 shows the comparisons between those who suffered LEA and those who did not. Subjects who underwent LEA had significantly longer pre-hospitalization duration of ulcer than those whose ulcers healed (63.9 ± 57.8 days vs. 35.0 ± 22.1 days; *P* <  0.001). They also suffered more peripheral artery disease (*P* <  0.001), had higher Wagner grades of ulcers (*P* <  0.001), more prevalent wound infection (P 0.039) and higher baseline levels of acute phase reactants (WBC and ESR).

**Table 1:** Demographic, clinical and laboratory characteristics of subjects who underwent lower extremity amputation and those who did not

Variable	Total (*n* = 336)	Amputation		*P* value
		Yes	No	
Age (years)	55.9 ± 12.5	56.8 ± 11.3	55.6 ± 13.1	0.391
Gender (male)	185 (55.1)	67 (36.2)	118 (63.8)	0.734
Diabetes type (type 1)	13 (3.9)	1 (7.7)	12 (92.3)	0.033
Diabetes duration (years)	8.5 ± 5.7	9.1 ± 5.7	8.1 ± 5.7	0.130
Ulcer duration (days)	45.2 ± 41.0	63.9 ± 57.8	35.0 ± 22.1	< 0.001
Ulcer duration (> 1 month)	237 (70.5)	111 (46.8)	126 (53.2)	< 0.001
Neuropathy	262 (78.0)	93 (35.5)	169 (64.5)	0.954
PAD	176 (52.4)	87 (49.4)	89 (50.6)	< 0.001
Wagner grades				
Grade 1	13 (3.9)	0 (0.0)	13 (100.0)	
Grade 2	57 (17.0)	3 (5.3)	54 (94.7)	
Grade 3	88 (26.2)	22 (25.0)	66 (75.0)	< 0.001
Grade 4	124 (36.9)	58 (46.8)	66 (53.2)	
Grade 5	54 (16.1)	36 (66.7)	18 (33.3)	
Presence of gangrene	178 (53.0)	94 (52.8)	84 (47.2)	< 0.001
Wound infection	258 (76.8)	99 (38.4)	159 (61.6)	0.039
Proteinuria	96 (28.6)	54 (44.6)	67 (55.4)	0.021
HbA1c (%)	9.6 ± 1.9	9..9 ± 1.8	9.4 ± 2.0	0.061
White cell count (cells/ml)	12.2 ± 6.0	13.5 ± 5.9	11.4 ± 5.9	0.003
ESR (mm/hour)	63.3 ± 34.8	75.4 ± 34.5	56.2 ± 33.0	< 0.001
Osteomyelitis	91 (27.1)	59 (64.8)	32 (35.2)	< 0.001
Anemia	180 (53.6%)	71 (39.4)	109 (60.6)	0.097
Renal impairment	66 (19.6%)	29 (43.9)	37 (56.1)	0.106

Data are in numbers (percentage) or means (± SD)

*PAD* peripheral artery disease, *HbA1c* glycated hemoglobin, *ESR* erythrocyte sedimentation rate

Table 2 shows univariate predictors of amputation among the study participants. Subjects with duration of ulcer more than 1 month were 10 times more likely to suffer LEA (95% CI 4.656–21.567; *P* <  0.001). Patients who had PAD (*P* <  0.001), Wagner grade 4 ulcer or higher (*P* <  0.001) and clinical wound infection (P 0.041) were approximately 4 times, 6 times and twice as likely to undergo LEA respectively.

**Table 2:** Univariate predictors of amputation

Variable	Amputation		*P* value	OR	95% C.I
	Yes n (%)	No n (%)			
Age ≥ 65 years	29 (37.2)	49 (62.8)	0.874	1.065	0.488–2.325
Gender (male)	67 (36.2)	118 (63.8)	0.734	1.081	0.689–1.695
Diabetes type (type 1)	1 (7.7)	12 (92.3)	0.065	0.145	0.019–1.127
Diabetes duration > 10 years	35 (40.7)	51 (59.3)	0.236	1.356	0.819–2.245
Ulcer duration > 1 month	111 (46.8)	126 (53.2)	**< 0.001**	10.021	4.656–21.567
Neuropathy	93 (35.5)	169 (64.5)	0.954	1.016	0.592–1.744
PAD	87 (49.4)	89 (50.6)	**< 0.001**	3.910	2.402–6.365
Ulcer grade (≥ 4)	94 (52.8)	84 (47.2)	**< 0.001**	5.953	3.544–10.002
Wound infection	99 (38.4)	159 (61.6)	**0.041**	1.806	1.024–3.183
Proteinuria	54 (44.6)	67 (55.4)	**0.021**	1.724	1.084–2.741
HbA1c < 7%	3 (17.6)	14 (82.4)	0.097	0.278	0.062–1.259
WBC > 10,000/μL)	78 (44.3)	98 (55,7)	**0.001**	2.412	1.413–4.118
ESR > 20 (mm/hour)	100 (38.9)	157 (61.1)	0.171	2.454	0.678–8.877
Osteomyelitis	59 (64.8)	32 (35.2)	**< 0.001**	5.879	3.462–9.983
Anemia	71 (39.4)	109 (60.6)	0.098	1.466	0.932–2.305
Renal impairment	29 (43.9)	37 (56.1)	0.100	1.585	0.916–2.743

*PAD* peripheral artery disease, *HbA1c* glycated hemoglobin, *WBC* white blood cells, *ESR* erythrocyte sedimentation rate. Note: *P* value entries in boldface denote statistical significance

The outcome of a multivariate regression analysis of all the factors entered at the univariate level is presented in Table 3. Ulcer duration more than 1 month was the strongest independent predictor of LEA after adjusting for all other variables (adjusted OR 10.29; 95% CI 4.055–26.132). This was followed by osteomyelitis (adjusted OR 5.62) and presence of PAD (adjusted OR 2.79).

**Table 3:** Multivariate predictors of amputation

	B	Adjusted O.R	*P* value	95% C.I.for OR	
				Lower	Upper
Ulcer > 1 month	2.331	10.3	< 0.001	4.055	26.132
PAD	1.025	2.8	< 0.001	1.520	5.107
Osteomyelitis	1.726	5.6	< 0.001	2.930	10.776

*PAD* peripheral artery disease

## Discussion

Besides death, lower extremity amputation is the most unpleasant consequence of diabetic foot ulcers, with far reaching medical and psycho-social impact. Outcome prediction in patients with DFU is likely to assist clinicians in prompt and appropriate decision making including individualization and optimization of available therapeutic options and resources. In spite of a growing burden of diabetes and diabetic foot ulcers in developing countries of Africa, published data on risk factors for LEA in this population are nonexistent.

In contrast to a Turkish study [19], we observed no significant association between age and LEA. Our observation is supported by many other studies which had demonstrated a lack of association between age and LEA in patients with DFU [20–22]. Although wound healing is generally slower in older than younger people, other factors beyond age such as nutritional status, vascular integrity and infection have been shown to play more dominant roles [23]. It is arguable that the younger age of our study population whose mean was 55.9 ± 12.5 years compared to that of the above cited study [19] with mean age of 62.65 ± 10.6 years might account for these divergent observations. However, in a large cohort of 2831 patients with DFU prospectively followed until healing or amputation, Gershater et al. [24] reported complete ulcer healing without amputation in 51% of patients older than 80 years and concluded that demographic variables including age, gender and duration of diabetes did not significantly predict amputation similar to our observations.

A longer duration of ulcer was strongly associated with LEA in this study. Our data revealed that ulcer longer than 1 month duration increased amputation risk ten folds even after adjustment for other confounding variables. Published studies that examined this variable on DFU outcome are scanty. Our observation is however in concordance with that of Uysal et al. [25] and may be explained by the fact that a longer duration of ulcer increases the probability of wound infection with resultant tissue necrosis. This notion is supported by Lavery et al. [26] who had demonstrated that ulcer duration more than 30 days was associated with a three-fold increase in wound infection. Both Wang et al. [20] and Tabur et al. [27] reported that wound infection increased amputation risk several folds. In confirmation of their findings, wound infection resulted in nearly twice the risk of amputation in our study, although this relationship became attenuated on multivariate analysis. However, bone infection/osteomyelitis persisted as an independent risk factor for LEA, increasing amputation risk by more than five times and emerging as the second strongest risk factor after duration of ulcer. This is consistent with many previous studies [19, 25, 28] and suggests that deep infections rather than infection per se plays more significant role on diabetic foot amputations.

Over three-quarter of our subjects presented with diabetic peripheral neuropathy (DPN), a well recognized risk factor for foot ulceration. However we did not observe a significant association between DPN and LEA contrary to the observations by Sadriwala et al. [28]. Our finding is supported by robust evidence as most researchers in this area have similarly documented a lack of association between DPN and LEA [20, 21, 24]. This raises the possibility that tissue denervation does not significantly impair wound healing in an offloaded foot. On the other hand, the presence of PAD independently increased the probability of LEA by nearly three-folds in our study, an observation that is consistent with most [19, 21, 24, 28] but a few [20] studies in this subject area. The process of wound granulation and healing requires adequate nutrient supply to the tissues and this is adversely affected in the presence of circulatory compromise [29]. Evidence has also recently emerged that PAD reduces tissue antibiotic concentration and increases proliferation of multidrug resistant microbes in diabetic foot wounds with resultant increase in the odds of amputation [30]. In a sub-analysis of the Eurodiale study, a multi-center observational study across 14 centers in Europe, Prompers et al. [31] reported that when the cohort was stratified according to presence or absence of PAD, wound infection emerged as the specific predictor of LEA in PAD patients only. This led the authors to conclude that the negative effect of infection on wound healing is restricted only to patients with PAD.

One factor that has emerged nearly always as a significant risk factor for LEA is ulcer grade [19, 20, 22, 27, 28]. This was confirmed in our study which showed that Wagner grade 4 ulcer and above increased LEA risk nearly six times. This observation is not surprising since ulcer severity including ulcer depth and probability of gangrene increases with higher Wagner grade. The presence of proteinuria emerged as a significant risk factor for LEA in this study. Proteinuria may be a sign of nephropathy in persons with diabetes. Although we did not quantify the degree of proteinuria nor measure serum albumin, our finding may be accounted for by hypoalbuminemia which has been shown to predispose to wound infection and delayed wound healing [32].

Research findings on the influence of glycemic control on amputation risk have been largely inconsistent. While a few authors have observed a significant association between the two [21, 28, 33], others have reported otherwise [22, 27]. Our study is one of those which failed to show any significant association between HbA1C and LEA. In fact in the study by Lee et al. [34], baseline HbA1c was even significantly higher in treatment success group than those who suffered LEA. These findings suggest that although poor glycemia is a potent risk factor for development of DFU, it is not necessarily a significant determinant of amputation in the patients.

Some authors have reported significant positive predictive value of acute phase reactants including WBC count, C-reactive protein and ESR on DFU-related LEA [35, 36]. However our observations contradict such findings. In agreement with the works of Pemayun et al. [21], we did not observe any significant effect of ESR on the odds of amputation. Increased white cell count showed promise of predicting LEA with unadjusted odds ratio of 2.4 but when adjusted for other covariates in multivariate analysis, no significant predictability was detected. Levels of CRP behaved the same way in a recent study in Saudi Arabia [20]. Prior to that, a Malaysian study had reported the inability of WBC, CRP and ESR to predict amputation in patients with DFU [37]. These observations support the argument that elevated levels of acute phase reactants in DFU patients is only secondary to other more important underlying pathologies such as limb ischemia, wound infection and osteomyelitis as suggested by Wang et al. [20]. Anemia is widely reported to impair wound healing [23, 38] and is expected to play a role in diabetic foot amputation. However, we did not observe any significant association between anemia and LEA, in concordance with reports of other investigators on this subject [21, 37]. This finding however contradicts the report by Yesil et al. [19] and Sun et al. [22] who observed significant associations between baseline hemoglobin concentrations and risk of LEA. Unlike other variables investigated against outcome in this study, anemia is usually quickly corrected with blood transfusion soon after hospitalization and this might have obscured its impact on wound healing and LEA in this study.

## Strengths and limitations

To our knowledge, the MEDFUN is the largest and the only multi-center study on diabetic foot ulcer not only in Nigeria but also in West-Africa; and the only study that extensively investigated the determinants of LEA in patients with DFU in this sub-region. Secondly, our study centers covered 4 out of the six geo-political zones of Nigeria. Since the remaining 2 geo-political zones share common characteristics with one or more of these 4 zones, our results are therefore largely generalizable as a true reflection of the burden of diabetic foot ulcer in Nigeria.

The limitations of this study however need to be highlighted. Firstly, distinction between type I and type II DM was made clinically as commonly practiced in most hospitals in Nigeria due to absence of facilities for routine anti-GAD 65 and plasma C-peptide assays. This may lead to misclassification of subjects by diabetes type. Secondly, our inability to conduct vascular imaging of the lower limbs for all the participants constitutes another limitation. Due to financial difficulties, only subjects with clinical suspicion of PAD underwent Doppler ultrasound. This has the potential of under diagnosing the condition due to observer bias. The ankle brachial index (ABI) which is a standard bedside screening tool for PAD could also not be utilized in this study due to technical problems associated with applying pressure cuffs on infected wounds and inflamed surrounding skin. Finally, each of the participating centers adopted its own DFU management protocol based on availability of manpower, and the decision to amputate or not was dependent on the clinicians at each center. It is not unlikely that this lack of uniformity might have affected the outcome of this study. This is also applicable to the clinical measurements which are prone to inter-observer bias and laboratory tests which might have been influenced by performance variations of diagnostic equipments at the different study centers. However, this lack of uniformity is common in studies of this nature, including the widely cited Eurodiale study [31] which was the largest multi-center diabetic foot ulcer study in Europe.

## Conclusion

This study has identified a number of important risk factors for LEA in subjects with DFU and these include duration of ulcer longer than 1 month, PAD, higher Wagner grade, wound infection, proteinuria and osteomyelitis. Interestingly, most of these identified predictors of LEA are factors that can be quickly assessed by the patient’s bedside. We believe that the findings in this study would assist general practitioners identify high risk patients who may benefit from early referral to specialist centers, and also guide foot care specialists in taking appropriate and timely clinical decisions. This may go a long way in reducing LEA rate in people with DFU in our environment.

## Data Availability

The datasets used and/or analyzed during the current study are available from the corresponding author on reasonable request.

## Abbreviations

DFU: Diabetic foot ulcer
DM: Diabetes mellitus
DPN: Diabetic peripheral neuropathy
ESR: Erythrocyte sedimentation rate
HbA1c: Glycated hemoglobin
LEA: Lower extremity amputation
MEDFUN: Multi-center Evaluation of Diabetic Foot Ulcer in Nigeria
PAD: Peripheral artery disease
T1DM: Type 1 diabetes mellitus
T2DM: Type 2 diabetes mellitus
WBC: White blood cell
